# Activation of Gq-Coupled Receptors in Astrocytes Restores Cognitive Function in Alzheimer’s Disease Mice Model

**DOI:** 10.3390/ijms24129969

**Published:** 2023-06-09

**Authors:** Evgenii Gerasimov, Ilya Bezprozvanny, Olga L. Vlasova

**Affiliations:** 1 Laboratory of Molecular Neurodegeneration, Peter the Great St. Petersburg Polytechnic University, Khlopina St. 11, 194021 St. Petersburg, Russia; evgeniigerasimov1997@gmail.com (E.G.); ilya.bezprozvanny@utsouthwestern.edu (I.B.); 2 Department of Physiology, UT Southwestern Medical Center at Dallas, Dallas, TX 75390, USA

**Keywords:** optogenetics, astrocytes, Alzheimer’s disease, long-term potentiation, behavioral tests, opto-α1-adrenoreceptor, Gq-coupled proteins, EAAT-2

## Abstract

Alzheimer’s disease (AD) is one of the most widespread neurodegenerative diseases. Most of the current AD therapeutic developments are directed towards improving neuronal cell function or facilitating Aβ amyloid clearance from the brain. However, some recent evidence suggests that astrocytes may play a significant role in the pathogenesis of AD. In this paper, we evaluated the effects of the optogenetic activation of Gq-coupled exogenous receptors expressed in astrocytes as a possible way of restoring brain function in the AD mouse model. We evaluated the effects of the optogenetic activation of astrocytes on long-term potentiation, spinal morphology and behavioral readouts in 5xFAD mouse model of AD. We determined that in vivo chronic activation of astrocytes resulted in the preservation of spine density, increased mushroom spine survival, and improved performance in cognitive behavioral tests. Furthermore, chronic optogenetic stimulation of astrocytes resulted in the elevation of EAAT-2 glutamate uptake transporter expression, which could be a possible explanation for the observed in vivo neuroprotective effects. The obtained results suggest that the persistent activation of astrocytes may be considered a potential therapeutic approach for the treatment of AD and possibly other neurodegenerative disorders.

## 1. Introduction

Glial cells are functionally divided into separate groups such as astrocytes, microglia, oligodendrocytes, etc. Astrocytes affect neuronal function in various ways, for example, by regulating the concentration of ions and neurotransmitters [1,2], releasing gliotransmitters that can act directly on neuronal receptors [3,4], and modulating neuronal excitability, synaptic transmission and plasticity [5,6]. Moreover, astrocytes establish direct connections with neurons by forming tripartite synapses [7,8]. Activation of astrocytes generates calcium waves [9,10,11,12] with the subsequent release of different gliotransmitters, neuromodulators and factors that modulate neuronal activity. These cells also release ATP, the main source of extracellular adenosine in the brain [13]. At the same time, it is worth considering the fact that a distinctive feature of some neurodegenerative disease is reactive gliosis with astrocytic morphological and functional changes [14,15,16,17].

Astrocytes are involved in the pathogenesis of Alzheimer’s disease (AD), causing inflammation and abnormal calcium signaling [18,19,20]. Alzheimer’s disease is the most common type of neurodegeneration. It is the most common type of neurodegeneration worldwide; however, there is no treatment nowadays for this illness. AD is characterized by the accumulation of Aβ-amyloid plaques [21,22,23] and tau tangles [24], by disrupted neuronal [25,26] and by astrocytic calcium homeostasis [27]. Many different approaches have been tested as pharmacological solutions to Alzheimer’s disease, such as targeting amyloid cascade, amyloid plaques and tau tangles. Nevertheless, these approaches have yielded little progress [28]. Thus, there is an urgent need to discover a new, effective drug therapy for Alzheimer’s disease. Potential promising approaches may also include astrocytic modulation [29,30,31,32,33].

One of the ways of precisely controlling or modulating astrocyte activity is the optogenetic method [5,34,35,36]. This allows scientists to regulate the activity of different cells through genetically constructed photosensitive channels—opsins [37,38,39,40,41]. In the current study, optogenetic modulation of astrocytic activity was performed via metabotropic OPTO-α1AR, the activation of which led to Gq pathway involvement [42]. We have shown earlier that the influence of the optogenetic activation of OPTO-α1AR, expressed in the hippocampal astrocytes, resulted in the elevation of neuronal activity, facilitation of fEPSP values in the hippocampal area and expression elevation of genes encoding various transcriptional factors [5,36]. In this study, the effect of the optogenetic modulation of astrocytes on long-term potentiation, mushroom spine density and fear conditioning test is shown in transgenic mice model of Alzheimer’s disease 5xFAD. The obtained data provide one more piece of evidence about the impact of astrocytes in the brain and show the possible ways of restoring synaptic deficit and strengthening long-term memory formation and preservation in the genetic model of Alzheimer’s disease in mice.

## 2. Results

### 2.1. Optogenetic Activation of Astrocytes Expressing OPTO-α1AR-EYFP in Hippocampus Restores LTP Formation in 5xFAD Mice

Optogenetic stimulation of astrocyte activity using OPTO-α1AR-EYFP leads to enhancement of hippocampal pyramidal neuron activity and strengthens fEPSP in acute hippocampal slices of mice, as was previously shown [5,35]. The evaluation of the possible positive effects on long-term potentiation formation was achieved using LTP recordings in hippocampal acute brain slices of 6-month-old mice of a wild type and a 5xFAD line as a genetic model of Alzheimer’s disease. LTP recordings were conducted 3 weeks after unilateral viral injection of the OPTO-α1AR-EYFP construct into the hippocampus. The extracellular and intracellular solutions were the same as those of standard ACSF. A twisted bipolar electrode was used for hippocampal Shaffer collateral simulation and as a conditioning protocol of high-frequency stimulation. Optogenetic stimulation of astrocytes (λ = 473 nm) was carried out after the application of baseline-level recording for 5 min with light 100 ms pulse parameters and an interval of 1 s [5] with the following HFS. In all the experiments, all the values were normalized to the mean value from baseline. The experimental group consisted of slices from the hemisphere where the injection of AAV2/5-GfaABC1D_Opto-a1AR_EYFP was performed and slices from another one formed a control group. Control slices were also subjected to optogenetic stimulation in order to evaluate possible phototoxic damage.

**Figure 1:**
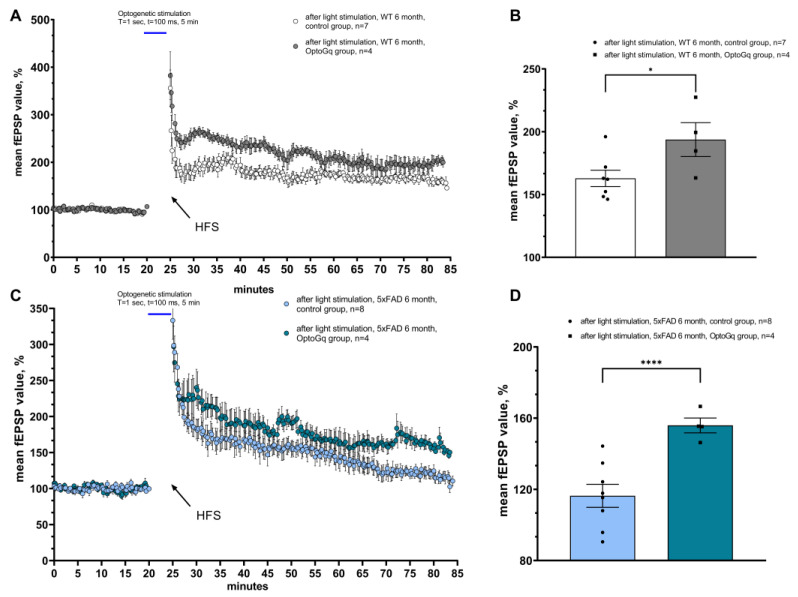
Optogenetic activation of OPTO-α1AR expressed in astrocytes restores LTP formation in 5xFAD mice model of Alzheimer’s disease. (**A**) The normalized average value of fEPSP value (%) before and after light stimulation of astrocytes expressing OptoGq in 6-month-old WT control and experimental group. (**B**) The normalized average fEPSP value after light stimulation of astrocytes expressing OptoGq in control and experimental groups of wild-type mice aged 6 months. The data are presented as an average ± SEM; *: *p* < 0.05 (Student’s *t* test). (**C**) The normalized average value of fEPSP value (%) before and after light stimulation of astrocytes expressing OptoGq in 6-month-old 5xFAD control and experimental group. (**D**) The normalized average fEPSP value after light stimulation of astrocytes expressing OPTO-α1AR in control and experimental groups of wild-type mice and 5xFAD mice aged 6 months. The data are presented as an average ± SEM; ****: *p* < 0.0001 (Student’s *t* test).

Firstly, in order to evaluate whether optogenetic stimulation of the Gq pathway in hippocampal astrocytes could affect LTP formation in normal conditions, experiments on WT mice hippocampal slices were conducted and their results are presented in Figure 1A, B. We have determined that optogenetic activation of metabotropic opsin OptoGq leads to strengthening long-term potentiation formation in the WT group of mice. In the experimental group of 4 mice, the mean value of the slope in 55th to 60th min of recording after high-frequency stimulation was 162.85 ± 6.51 (*n* = 7) in the control group and 193.70 ± 13.49 (*n* = 4) in the experimental group. A significant difference was observed between these groups in the mean slope value in the last 5 min of recording (*p* = 0.0436, Student’s *t* test). This finding could be explained by NMDA agonist excretion from astrocytes and be involved in enhanced LTP formation. Further, following the presented scheme of the experiment, recordings were conducted of long-term potentiation on acute slices from 6-month-old 5xFAD mice (4 mice). It has been widely shown that 5xFAD mice are not able to undergo long-term stable changes in normal conditions [43,44,45,46]. Optogenetic stimulation of OPTO-α1AR-EYFP expressed in astrocytes restores LTP formation deficit in 5xFAD mice (Figure 1C,D). The mean values of the last 5 min of the slope were measured in the control group (116.34 ± 6.47 (*n* = 8)) and were significantly higher in the experimental group (156.43 ± 4.17 (*n* = 4)) (*p* = 0.002, Student’s *t* test).

**Figure 2:**
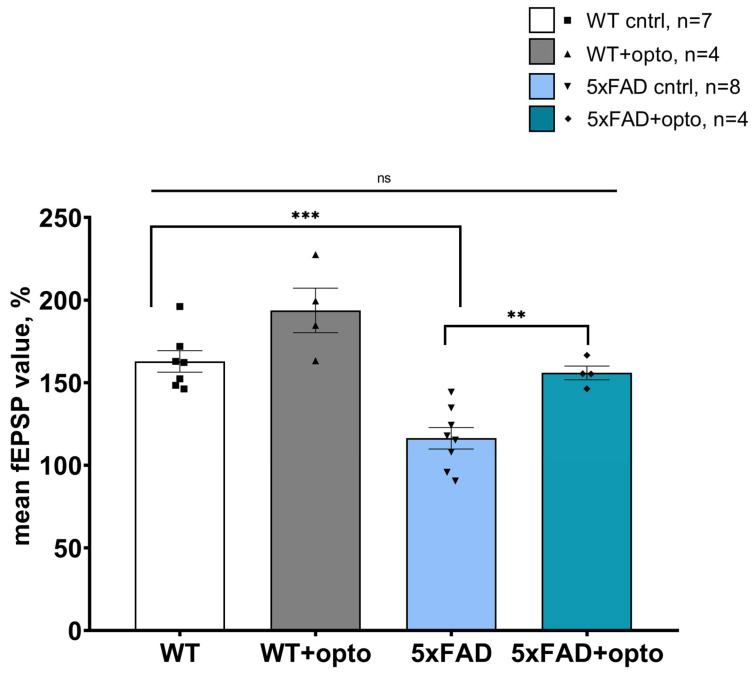
The normalized average fEPSP value after light stimulation of astrocytes expressing OPTO-α1AR in control and experimental groups of wild-type mice and 5xFAD mice aged 6 months. The data are presented as an average ± SEM; ns: there are no significant differences; **: *p* < 0.01, ***: *p* < 0.001 (Two-factor analysis of variance (ANOVA) followed by the Tukey test).

A comparison of all mean values from WT and 5xFAD groups is presented in Figure 2. As was expected, there was a crucial deficit in LTP formation in control slices from 5xFAD mice (162.85 ± 6.51 (*n* = 7) for the WT control group and 116.34 ± 6.47 (*n* = 8), *p* < 0.0001, two-factor analysis of variance (ANOVA) followed by the Tukey test). Additionally, there was no differences between the mean value of the control group of WT mice and the mean value of the experimental group of 5xFAD mice (162.85 ± 6.51 (*n* = 7) and 156.43 ± 4.17 (*n* = 4) correspondingly, *p* = 0.90, two-way ANOVA followed by the Tukey test). However, there was a valuable difference in comparison of wild-type mice of the experimental group and the experimental group of 5xFAD mice (193.70 ± 13.49 (*n* = 4) and 156.43 ± 4.17 (*n* = 4) correspondingly, *p* = 0.011). These phenomenological data could provide the possibility of assuming that positive modulators of NMDA receptors can be secreted by astrocytes after optogenetic stimulation of Gq pathways in astrocytes, meaning that long-term changes in the WT experimental group are higher than those in the 5xFAD experimental group. Nevertheless, stable amplification of fEPSP in the experimental group of 5xFAD mice might impact on the cognitive function of these mice in behavioral tests.

### 2.2. Modulation of Astrocytic Activity through Gq Pathway Beneficially Impacts on Cognitive Function of 5xFAD Mice

**Figure 3:**
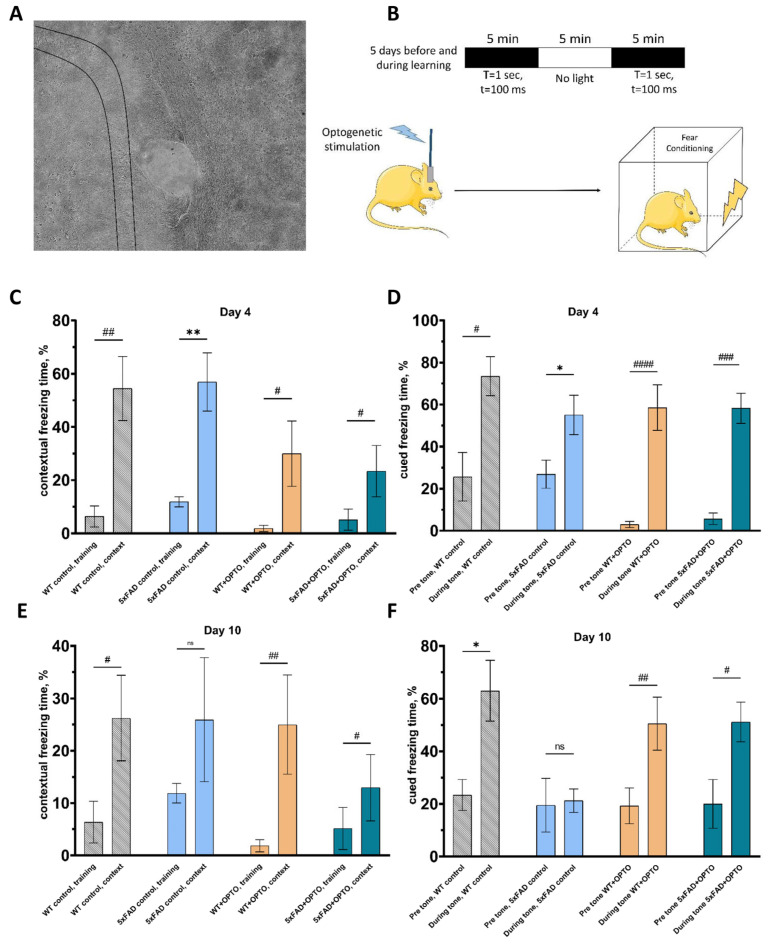
Beneficial impact of OPTO-α1AR optogenetic activation on cognitive function of transgenic 5xFAD mice. (**A**) Location of fiber for optogenetic stimulation above hippocampal neurons, the borders of which are highlighted by black lines, 10× magnification. (**B**) Protocol for fear conditioning testing for all groups of mice. (**C**) Total context freezing time (%) of all control and experimental groups of WT and transgenic 5xFAD mice in the FC test on the day 4. (**D**) Total cued freezing time (%) of all control and experimental groups of WT and transgenic 5xFAD mice in the FC test on day 4. (**E**) Total context freezing time (%) of all control and experimental groups of WT and transgenic 5xFAD mice in the FC test on day 10. (**F**) Total cued freezing time (%) of all control and experimental groups of WT and transgenic 5xFAD mice in the FC test on the day 10. All the data are presented as an average ± SEM; ns: there are no significant differences; *: *p* < 0.05, **: *p* < 0.01 (Student’s *t* test); #: *p* < 0.05, ##: *p* < 0.01, ###: *p* < 0.001, ####: *p* < 0.0001 (Mann–Whitney test). Picture B is crafted with the help of https://smart.servier.com/, accessed on 16 September 2022.

To evaluate whether optogenetic stimulation of OPTO-α1AR expressed in hippocampal astrocytes could influence the cognitive functions of the mice line 5xFAD, fear conditioning (FC) testing was performed. The hippocampus plays one of the most important roles in the process of learning and, being a part of a limbic system, has a connection with the amygdala, the role of which corresponds to a fear center. In these experiments, two groups were formed: the first one included control WT and 5xFAD mice without any exposure in order to provide an overview of phenotypical differences between WT and 5xFAD mice 7 months old. The second one consisted of mice with a unilateral injection of AAV2/5-GfaABC1D_Opto-a1AR_EYFP into the hippocampus for a group of 5xFAD mice and sterile saline injection into the hippocampus for the WT group. After surgery, optical fiber was fixed above the hippocampus (Figure 3A). Three weeks later, optogenetic stimulation was applied within 5 days before testing and each learning day with the protocol described in Figure 3B. Fear conditioning testing was carried out via a standard protocol with an auditory signal as a conditional stimulus. In short, on the first day and the second day, mice habituated to the fear conditioning chamber environment. The third day of the test was associated with the development of an associated fear in mice of an unconditional stimulus. Then, there was a one-time training of associated fear, where an unconditional electric current stimulus was supplied immediately after the conditional stimulus in the form of a previously unfamiliar sound. Then, the training was repeated, while the level of fear of returning to the sound was recorded. On the next day, day 4, the mouse was placed in the same test cage as when learning associated fear, but no more training was conducted. Instead, the level of fear was recorded for a specific situation associated with a previous negative experience (Figure 3C). Next, changes were made to the test cage to create a new environment that was unfamiliar to mice. Afterward, the sound was turned on, and on the third day, the level of fear associated with the conditional signal was registered (Figure 3D). A week later, on day 10—the second day of testing—the same sequence was repeated to determine long-term memory that is presented for both groups in Figure 3E,F.

On day 4–24 h after training, the level of freezing was recorded in order to determine the formation of the contextual memory in the old environment where training was performed and the associative memory to the sound in novel conditions (Figure 3C). On day 4, all the groups of mice showed a stable presentation of the contextual memory expressed in freezing time (%): WT control group (training: 6.35% ± 3.99% vs. context: 54.45% ± 11.32%, *n* = 8, *p* = 0.0011, Mann–Whitney test), transgenic 5xFAD mice (training: 11.88% ± 1.87% vs. context: 56.89% ± 10.13%, *n* = 6, *p* = 0.0023, Student’s *t* test), experimental group of WT mice (training: 1.70% ± 1.29% vs. context: 33.54% ± 12.57%, *n* = 9, *p* = 0.0117, Mann–Whitney test) and an experimental group of transgenic 5xFAD mice (training: 5.16% ± 4.01% vs. context: 23.38% ± 9.64%, *n* = 7, *p* = 0.0152, Mann–Whitney test). The same tendency was also seen in the validation of the cued memory on day 3: WT control group (pre-tone: 25.66% ± 10.80% vs. during tone: 71.64% ± 9.79 %, *n* = 8, *p* = 0.0059, Mann–Whitney test), transgenic 5xFAD mice (pre-tone: 26.87% ± 6.71% vs. during tone: 55.10% ± 9.38%, *n* = 6, *p* = 0.0343, Student’s *t* test), experimental group of WT mice (pre-tone: 2.02% ± 1.02% vs. during tone: 59.50% ± 11.58%, *n* = 9, *p* < 0.0001, Mann–Whitney test) and experimental group of transgenic 5xFAD mice (pre-tone: 5.71% ± 2.95% vs. during tone: 58.26% ± 7.74%, *n* = 7, *p* = 0.0006, Mann–Whitney test) (Figure 3D). All the groups of mice performed a stable level of freezing, which was correlated with normal early memory representation in fear conditioning testing.

A week later, on day 10, context memory representation was stable in the control group of wild-type mice (training: 6.35% ± 3.99% vs. context: 26.24% ± 8.15%, *n* = 8, *p* = 0.0468, Mann–Whitney test) and in the experimental group of WT mice (training: 1.70% ± 1.29% vs. context: 28.11% ± 11.69%, *n* = 9, *p* = 0.0062, Mann–Whitney test). The same results were also observed in the cued freezing memory validation in the control group of WT mice (pre-tone: 23.41 ± 5.89; during tone: 63.04 ± 11.53, *n* = 6, *p* = 0.0085, Student’s *t* test) and in the experimental group of WT mice where saline was injected and optostimulation was applied (pre-tone: 19.27 ± 6.76; during tone 50.55 ± 10.06, *n* = 9, *p* = 0.0056, Mann–Whitney test).

As has been widely shown, 5xFAD mice are not able to form long-term memory in fear conditioning testing [47,48,49]. Control transgenic 5xFAD mice have shown an absence of any changes in the context freezing time that is correlated to long-term memory formation failure (training: 11.88% ± 1.87% vs. context: 25.93% ± 11.83%, *n* = 6, *p* = 0.2678, Student’s *t* test). The same results for the group of control 5xFAD mice were obtained for freezing time in response to conditional stimuli (19.53 ± 9.33 and 21.22 ± 4.07 correspondingly, *n* = 6, *p* = 0.8835, Student’s *t* test). These data correspond to failure in long-term memory formation in the transgenic mice model of Alzheimer’s disease. In contrast, the experimental group of transgenic 5xFAD mice has shown a stable representation of the context memory (training: 5.16% ± 4.01% vs. context: 12.95% ± 6.33%, *n* = 7, *p* = 0.0478, Mann–Whitney test) and the cued memory (pre-tone: 20.02% ± 9.28%; during tone: 51.17% ± 7.54%, *n* = 7, *p* = 0.038, Mann–Whitney test). By using the fear conditioning behavioral test, a great increase was observed in long-term memory formation expressed in freezing time as a response to context and conditional stimuli.

These data correlate to the strengthening of synaptic plasticity that was shown in LTP experiments in Section 2.1, so, we assume that the same phenomena might be the basis of significant impact on memory formation and its consolidation in the 5xFAD mice model of Alzheimer’s disease. It should be noted that astrocytes secrete a wide spectrum of neuro- and gliotransmitters, neuromodulators and factors which, possibly being excreted due to optogenetic stimulation of OPTO-α1AR, might have a very complex effect on neuronal functioning, synaptic plasticity and long-term memory changes. The last ones are tightly connected with dendritic spines status and morphology, especially those located in the hippocampus [50,51,52]. It has been shown that dendritic spines, in particular mushroom spines, suffer from Alzheimer’s disease progression and that their loss is one of the hallmarks of most neurodegenerative disorders [53,54,55]. Therefore, we suggested that improvements in long-term memory formation could be explained by morphological changes and the elevation of spine number in the hippocampus of transgenic mice model of Alzheimer’s disease (see the next chapter).

Further, the Morris water maze (MWM) test was performed for the determination of an astrocytic OPTO-α1AR effect on spatial memory formation and learning in wild-type mice and transgenic 5xFAD mice [56,57,58]. As was expected [59,60,61,62,63], transgenic 5xFAD mice from the control group showed a learning deficit in the MWM test that was expressed in the absence of statistical changes between the first (day 1) and the fourth training days (day 4) in the value of latent time of platform finding (seconds) (85.21 s ± 5.39 s and 79.49 s ± 9.04 s correspondingly, 18, *n* = 6, *p* = 0.3485, Student’s *t* test), while control wild-type mice saw a significant reduction of latent time value between day 1 and day 4 (74.24 s ± 10.52 s and 26.76 s ± 8.31 s correspondingly, *n* = 6, *p* < 0.0001, Student’s *t* test). In contrast to the control WT group, the experimental group of wild-type mice did not show any changes in latent time (day 1: 53.15 s ± 12.60 s vs. day 4: 49.85 s ± 11.18 s, *n* = 8, *p* = 0.3445, Student’s *t* test). We did not find it reasonable to show the obtained data due to the poor results of experimental groups in the test. As a reasonable explanation, it can be assumed that fiber interferes with the normal processes of spatial orientation and cannot be used in the Morris water maze testing without any external amendments.

### 2.3. Optogenetic Stimulation of OPTO-α1AR Expressed in Astrocytes Increase Mushroom Spine Density in 5xFAD Mice

**Figure 4:**
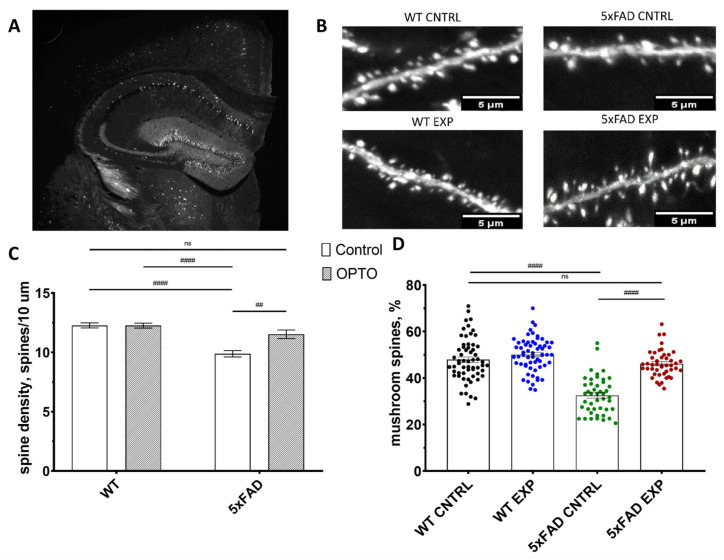
Modulation of astrocytic activity by OPTO-α1AR leads to an increase in mushroom spine density in 5xFAD. (**A**) Hippocampus of a WT mouse from WT m line, magnification 4×. (**B**) Representative images of secondary dendrites with spines from all groups, scale bar is shown in the picture, magnification 100×. (**C**) Spine density of secondary dendrites from hippocampal neurons per 10 µm in experimental and control groups of 6-month-old WT and 5xFAD mice. (**D**) Percent of mushroom spines on secondary dendrite of hippocampal neurons in experimental and control groups of WT and 6-month-old 5xFAD mice. Each point shows individual value for each dendrite. The data are presented as an average ± SEM; ns: there are no significant differences, ^##^: *p* < 0.01, ^####^: *p* < 0.0001 (Kruskal–Wallis test with multiple comparisons by Dunn’s test).

The determination of the possible influence of the optogenetic stimulation of astrocytes expressing metabotropic photosensitive constructs on the density and morphology of dendritic spines was performed in the next series of experiments. Similarly, like in the previous chapter, fiber was implanted above the hippocampal neurons for optogenetic stimulation in the head of mice (Figure 3A). For this purpose, we used 6-month-old mice of WT-mline and 5xFAD-mline that expressed GFP in the neurons of the brain. A 5xFAD-mline mice experimental group was presented as slices from the hemisphere where AAV2/5-GfaABC1D_Opto-a1AR_EYFP was injected and optogenetic stimulation was applied through an optofiber, control group consisted of slices from opposite hemisphere. For WT-mline mice saline was injected in the experimental group and light stimulation was applied and a control group was also formed from the opposite hemisphere’s slices. Optogenetic stimulation was provided 3 weeks after the viral or saline injection and lasted for 5 days with the protocol of 100 ms light pulses with 1 s intervals (5 min on–5 min off–and 5 min on, the same as was for fear conditioning testing) once a day. The density and spine morphology of spines were evaluated in the fixed 36 µm hippocampal sagittal slices (Figure 4B). Secondary dendritic spines were taken for analysis as the most stable characteristics of the density and distribution of the mushroom spine type.

Spine density (spines/10µm) was 9.82 ± 0.27 in the 5xFAD control group (*n* = 45 from 3 mice) and 12.27 ± 0.21 in the WT control group (*n* = 60 from 4 mice) (*p* < 0.0001, Kruskal–Wallis test with multiple comparisons by Dunn’s test). Such a strong decline in spine density was also observed previously in our lab [53,64]. However, we did not observe any significant differences between the control and experimental group of wild-type mice (12.27 ± 0.21 and 12.25 ± 0.22 correspondingly, *p* > 0.9999, Kruskal–Wallis test with multiple comparison by Dunn’s test). These data show that fiber and light pulses did not affect spine density and morphology in any way. Consequently, no negative effect on neuronal condition post-operation was determined. Optogenetic stimulation of the astrocytic Gq pathway led to a significant enlargement of spine density value of hippocampal dendrites in the 5xFAD mice model (5xFAD control group: 9.82 ± 0.27 (*n* = 45 from 3 mice) vs. 5xFAD experimental group: 11.52 ± 0.30 (*n* = 45 from 3 mice), *p* = 0.0027, Kruskal–Wallis test with multiple comparisons by Dunn’s test) that is presented in Figure 4C. Moreover, spine density in the 5xFAD experimental group did not differ from the WT one (11.52 ± 0.30 and 12.25 ± 0.22 correspondingly, *p* = 0.4386, Kruskal–Wallis test with multiple comparisons by Dunn’s test).

Further, the mushroom spine percentage was calculated from the same dataset. As was expected, there was a large difference in this parameter between the wild-type control group (48.88% ± 1.2%) and the 5xFAD control group (32.52% ± 1.19%) (*p* < 0.0001, Kruskal–Wallis test with multiple comparisons by Dunn’s test). The same correlation of positive optogenetic impact on mushroom spine restoration in 5xFAD mice was observed (5xFAD control: 32.52% ± 1.19% vs. 5xFAD-exp 46.14% ± 0.9%, *p* < 0.0001, Kruskal–Wallis test with multiple comparisons by Dunn’s test). Additionally, the percentage of the mushroom spine in 5xFAD-exp group recovered to control values in WT both groups (5xFAD-exp: 46.14% ± 0.9% vs. WT-cntrl: 48.88% ± 1.2%, *p* > 0.9999 and 5xFAD-exp: 46.14% ± 0.9% vs. WT-exp: 50.07% ± 1.0%, *p* = 0.1080, Kruskal–Wallis test with multiple comparisons by Dunn’s test). These data showed the positive influence of the optogenetic regulation of astrocytic activity by metabotropic OPTO-α1AR opsin on secondary dendritic spine density and the percentage of mushroom spines in the 5xFAD mice model of Alzheimer’s disease. These phenomenological data of spine recovery after optogenetic activation can explain the results of fear conditioning testing that were shown earlier in this paper.

### 2.4. Chronic Optogenetic Stimulation of Hippocampal Astrocytes Doesn’t Transform Them into Reactive Ones

To examine the effect of repetitive optogenetic stimulation of hippocampal astrocytes expressing Opto-a1AR on the possible changes in protein expression profile, Western blot method was used. To understand whether optostimulation for 5 days (as for experiments described above) might have a negative influence on astrocyte state expression of the major marker of reactive astrocytes, we observed the hippocampi of FVB 3- to 5-month-old mice. GFAP—glial fibrillary acidic protein—is a major and often-used marker of reactive (pathological) astrocytes [65]. In the conditions of brain trauma [66,67], seizure [68,69] and different neurodegenerative disorders [70], elevation of GFAP expression is observed. The experimental group consisted of the hippocampus from 3.5-month-old FVB female mice (*n* = 4) where Opto-a1AR was expressed and light stimulation was addressed via fiber, the control group consisted of the opposite hemisphere. To evaluate the possible effect of light stimulation on the expression of investigated proteins, a control group was created. In it, saline was injected into the hemisphere of mice (*n* = 4), light was delivered in the same way, and the control group was performed with the opposite hemisphere.

**Figure 5:**
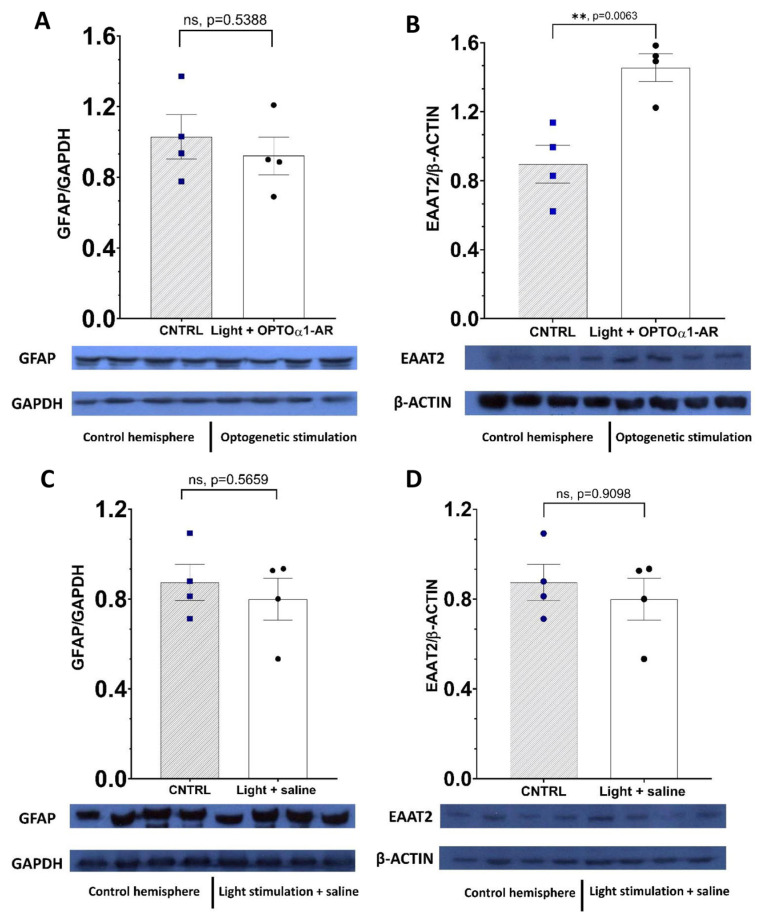
Optogenetic stimulation of astrocytic G-coupled protein did not lead to changes in the levels of GFAP expression but elevated expression of EAAT-2. (**A**) Quantitative analysis of the expression of GFAP level in the experimental group of mice. (**B**) Quantitative analysis of the expression of EAAT-2 level in the experimental group of mice. (**C**) Quantitative analysis of the expression of GFAP level in the control group of mice. (**D**) Quantitative analysis of the expression of EAAT-2 level in the control group of mice. *N* = 4 mice (each band represents hippocampal lysate for individual mice hemisphere). The data are presented as an average ± SEM; ns: there are no significant differences, **: *p* < 0.01.

It was determined that chronic optogenetic stimulation did not alter levels of GFAP expression in either the experimental group (*p* = 0.5388, *n* = 4, Student’s *t* test) (Figure 5A) or the control group (*p* = 0.5659, *n* = 4, Student’s *t* test) (Figure 5C). It provided information about the absence of a negative effect on astrocyte status and chronic optogenetic stimulation that did not transform normal astrocytes into reactive ones. For further examination of the chronic optogenetic stimulation effect on protein expression, levels of EAAT-2 were observed. EAAT-2 is a glutamate transporter that is predominantly expressed in the astrocytes [71]. By means of Western blot analysis, it was shown that in the experimental group of hemispheres level of EAAT-2 was elevated in comparison to control ones (*p* = 0.0063, *n* = 4, Student’s *t* test) (Figure 5B). Moreover, we found no changes in the control group in the expression of this transporter (*p* = 0.9098, *n* = 4, Student’s *t* test) (Figure 5D).

## 3. Discussion

Previously, we determined that the stimulation of astrocytes expressing the OptoGq metabotropic construct led to an increase in the number of spontaneous excitatory postsynaptic currents (sEPSC) in the pyramidal neurons of the hippocampus, as well as to the potentiation of the fEPSP values in the acute brain slices of the hippocampus [5]. Moreover, the activation of Gq-coupled receptors in astrocytes enhanced early immediate gene expression [36]. Based on this evidence, the current study showed the effect of optogenetic stimulation of the metabotropic opsin OPTO-α1AR expressed in astrocytes on the formation of long-term potentiation in mice with the genetic model of Alzheimer’s disease known as 5xFAD. It was estimated that the stimulation, lasting 5 min, of metabotropic opsin in astrocytes with optogenetic excitation parameters T = 1 s t = 100 ms led to a strengthening of synaptic plasticity in acute slices of the hippocampus. This was true in both the experimental group of wild mice and the experimental group of transgenic mice. The results of behavioral tests presented in this paper confirm the results obtained in the LTP experiments, in which an increase in synaptic plasticity was recorded in 5xFAD mice. One of the explanations for the effect of potentiation of synaptic plasticity, and, as a consequence, the recovery of values of freezing time in 5xFAD mice in the fear conditioning behavioral test, may be the release of neurotransmitter glutamate after the optostimulation of astrocytes [72,73,74,75]. This fact could explain the increase in LTP levels in the control and experimental groups of wild-type mice. However, increased glutamate levels are observed in Alzheimer’s disease. It is possible that glutamate is released by astrocytes via the modulation of metabotropic opsin synaptically, which has a positive effect on the condition of neurons and their functioning. It can be assumed that there is no extrasynaptic excitement due to glutamate spillover, as a result of which a neurotoxic effect is observed, expressed in excitotoxicity and neuronal death [76]. The effect of a persistent increase in the normalized slope value after the optogenetic modulation of astrocyte activity in wild-type mice could also be explained by the release of D-serine by astrocytes. It is an endogenous ligand to the glycine site of the NMDA receptor [77]. This coagonist regulates synaptic plasticity in the hippocampus of mice. One likely result is that there is a significant difference between the control and experimental group of wild-type mice in our study. However, the question remains as to whether it is possible to explain the recovery effect of the formation of long-term potentiation in 5xFAD mice via only the excretion of D-serine from astrocytes. The level of D-serine in blood plasma in patients with Alzheimer’s disease decreases in comparison with the control group [78]. It can also be found that the level of both L-serine, a precursor of D-serine, and D-serine in the brain, significantly decreases in mice of the 3xTg-AD mice line [79]. Moreover, the administration of this NMDA receptor agonist leads to recognition and social memory improvements [80]. At the same time, assuming the release of D-serine by astrocytes, there is a possibility of hyperactivating NMDA receptors, which already have an increased level of activity due to αβ-amyloid toxicity and elevated levels of Ca2+ [81,82]. In combination with D-serine, a wide range of only excitatory, and, probably, inhibitory neurotransmitters and neuromodulators are released [83,84,85,86]. These together cause a positive effect on the restoration of the formation of long-term potentiation and improvement of the cognitive functions of transgenic mice of the 5xFAD line. The cognitive decline of the 5xFAD mice is a well-established fact [87,88,89]. For the evaluation of the effect of astrocytic Gq-coupled receptors activation, a fear conditioning behavioral test was performed. The hippocampus plays an essential role in the process of learning and, being a part of a limbic system, it has a connection with the amygdala, which role corresponds to a fear center. There are several paradigms of FC testing [90,91]. Here, we used an auditory one because it corresponds well with changes observed in the downstream of Alzheimer’s disease and other neurodegenerative diseases [92,93]. We used optogenetics to evaluate the positive effect of hippocampal astrocytic OPTO-α1AR activation that was expressed in the increase in the context and cued freezing time. These results could be explained via spine preservation, especially of mushroom spines, in the 5xFAD transgenic mice, an issue which will be discussed further in this section. However, the absence of any effect was shown in the Morris water maze test, which is connected to spatial memory and the functioning of the hippocampus [94]. The most probable explanation for the failure of the test due to the lack of learning in both experimental groups of WT and transgenic 5xFAD, is the weight of fiber which is fixed to the head of the mouse. Hereinafter, it should be examined whether this statement is correct. Nowadays, there are ways to stimulate distinct types of cells via chemogenetic methods [95,96,97,98]. Using this technique for the stimulation of hippocampal astrocytes, researchers can obtain valuable results that enlarge knowledge about Gq pathway simulation of hippocampal astrocytes in mice models of Alzheimer’s disease.

It was also shown that optogenetic stimulation of OPTO-α1AR, expressed in astrocytes, has a positive effect on the density of dendritic spines and, in particular, on their morphology, which is expressed in the recovery of the number of mushroom spines in hippocampal neurons in the mouse line of Alzheimer’s disease 5xFAD. Dendritic spines (contacts between two neurons) play integral roles in the functioning of various areas of the brain [99,100]. Due to their high significance, the loss of spines leads to pathological processes, weakening the LTP formation and violations of memory processes [101,102]. The mechanisms of restoring the density and number of dendritic spines presented in this article in 5xFAD mice can be associated with the normalization of neuronal activity after optogenetic modulation of astrocytes. The mechanisms described earlier also likely play a role in the basis of an increase in the number of mushroom spines in transgenic mice. The modulation of astrocytes allows us to regulate the composition of the extracellular environment of the synapse region and the intracellular environment of neurons [7]. In this regard, directed modulation of astrocytic activity can directly affect the state of synapses, affecting their morphology.

The results of Western blot analysis have shown the absence of any negative changes in astrocyte state after chronic optogenetic stimulation of astrocytes in vivo. GFAP levels remain the same in the hippocampal region of the experimental and the control group of mice. This provides a piece of evidence about the influence of the chronic optostimulation of astrocytes on their state—astroglial cells do not turn into their reactive form with daily exposure on Gq-coupled proteins. Nevertheless, optogenetic stimulation of astrocytes expressing OPTO-α1AR leads to a significant increase in the expression of EAAT-2 protein (excitatory amino acid transporter 2). Its expression is decreased in patients with AD [103,104,105], furthermore, overexpression of EAAT2 has been shown to protect neurons from αβ-amyloid damage to neurons in APP transgenic mice [106]. EAAT2 plays an essential role in glutamate uptake, protecting neurons from the excessive effects of that neurotransmitter. Alzheimer’s disease is characterized by elevated levels of glutamate [107,108] which triggers NMDAR’s hyperfunction and excitotoxicity [109,110,111]. A possible explanation of the beneficial impact of optogenetic stimulation of astrocytic OPTO-α1AR might be an elevated level of EAAT2 expression, which leads to the uptake of excessive glutamate and decreased glutamate toxicity in neurons. This transporter has long being discussed as a potential therapeutic target for Alzheimer’s disease [105,112] and this study provides one of the ways of overexpression of EAAT2. We assume that long-term effects on synaptic spines are likely to be significantly influenced by changes in EAAT-2 expression levels in astrocytes.

It should be noted that all of the above-mentioned possible mechanisms underlying the positive effect of optogenetic stimulation of metabotropic opsin in astrocytes should be verified, thereby confirming or refuting the assumptions made. Finding the exact mechanisms and targets responsible for the positive effect of modulation of astrocyte activity is an important task as it can become a new therapeutic target for correcting the work of brain structures susceptible to Alzheimer’s disease.

## 4. Materials and Methods

### 4.1. Animals

The breeding colony of 5xFAD mice (B6SJLF1/J background, Jackson Labs: strain #006554, Bar Harbor, ME, USA) was established and maintained in a vivarium with 4–5 mice per cage and a 12 h light/dark cycle in the animal facility. Transgenic mice from this line in the paper are called transgenic, and the WT mice from the same breed—B6 mice—are called wild-type mice (WT). Mice from this line were used for long-term potentiation experiments and fear conditioning behavioral tests. For experiments on establishing hippocampal dendritic spine density and morphology, crossbreeding between 5xFAD and M-line (Thy1-GFP line M, Jackson Labs: stock #:007788) mice was performed. A breeding colony of Albino inbred mice (FVB/NJ) were kept in the same conditions (Jackson Labs, strain #001800). These mice were used only to obtain hippocampal lysates after optogenetic stimulation experiments in vivo with subsequent Western blot analysis. All procedures were approved by principles of the European convention (Strasburg, 1986) and the Declaration of International Medical Association regarding the humane treatment of animals (Helsinki, 1996) and approved by the Bioethics Committee of the Peter the Great St. Petersburg Polytechnic University at St. Petersburg, Russia (Ethical permit number 2-n-b from 25 January 2021).

### 4.2. Viral Constructs Delivery via Stereotaxic Surgery

A viral construct AAV2/5_GfaABC1D_Opto-a1AR_EYFP was delivered to the hippocampus by means of stereotaxic surgery. Injections of viral constructs were performed using a stereotaxic device (68001, RWD Life Science, Guangdong, Shenzhen, China), a syringe with a thin needle (84,853, 7758-02, Hamilton, Reno, NV, USA), as well as a heated mat and a temperature controller (69,002, RWD Life Science, Guangdong, Shenzhen, China). Surgery was carried out under the anesthesia of the animals by isoflurane 1.5–2.0% anesthetizing. After performing a control check of the depth of anesthesia in the animal, the viral construct was administered according to the standard protocol [113] at the following coordinates: AP − 2.1, DV − 1.8, ML + 2.1, with a volume of 1.5 µL at a rate of 0.1 µL/min with 15 min waiting after virus delivery without lifting a syringe.

### 4.3. Long-Term Potentiation Recording

Transcardial perfusion was performed with saturated carbogen (95% O_2_/5% CO_2_) 0–2 °C normal ACSF solution (124 mM NaCl, 2.5 mM KCl, 1.25 mM NaH_2_PO_4_, 24 mM NaHCO3, 5 mM HEPES, 12.5 mM D-glucose, 2 mM CaCl_2_, 2 mM MgSO_4_) and decapitation was performed. Horizontal slices of the brain with a thickness 400 µm were obtained by microtome for the recording of extracellular field potentials (Leica VT1200S (Leica Biosystems Division of Leica Microsystems Inc., Buffalo Grove, IL, USA)) from 6-month-old mice and maintained in a 0–2 °C solution of normal ACSF with carbogen saturation. The hippocampus was isolated from each slice and incubated in HEPES-ACSF solution (92 mM NaCl, 2.5 mM KCl, 1.25 NaH_2_PO_4_ 30 mM NaHCO_3_, 20 mM HEPES, 25 mM D-glucose, 2 mM thiourea, 5 mM Na-ascorbate, 3 mM Na-pyruvate, 2 mM CaCl_2_, 2 mM MgSO_4_) and saturated with carbogen with a controlled temperature (32–35 °C) for 15 min, after which the slices were incubated at room temperature of 23–25 °C. All the reagents mentioned above were obtained from Sigma-Aldrich, St. Louis, MO, USA. After an hour of incubation at room temperature, hippocampal slices were used to perform long-term potentiation experiments. FEPSP were recorded by the glass pipette electrode, containing normal ACSF solution with resistance of 250–400 kΩ in the CA1 area of stratum radiatum. During recording, fEPSP were filtered at 800 HZ filtering. No more than two recordings were taken from each hemisphere of the mice. Each group was presented as 4 mice. Optogenetic light stimulation was performed by means of the blue LED (LED4D067, 470 nm, Thorlabs Inc., Newton, NJ, USA), with maximum intensity of 35 mW mm−2 and with a maximum photo flux of 250 mW. After optogenetic stimulation was performed for slicing, high-frequency stimulation was applied with then following characteristics: 100 pulses for 1 s with a two-time repetition after 20 s. Then, 60 min-potentiated fEPSP were recorded.

### 4.4. Optogenetic Stimulation of Astrocytic Activity In Vivo

For optogenetic stimulation of hippocampal astrocytes that were expressing OPTO-α1AR metabotropic opsin, a light delivery system was used. It contained a light source (DC2100—High-Power, 1-Channel LED Driver with Pulse Modulation, 2 A, 24 V, ThorLabs, Newton, NJ, USA), and a fiber-coupled LED with 470 nm (M470F1—Blue (470 nm) Fiber-Coupled LED, SMA, 1000 mA, 8.0 mW (Min), ThorLabs, Newton, NJ, USA). Fiber Optic Patch Cables with Ferrule Ends (ThorLabs, Newton, NJ, USA), long ceramic ferrule (CF340-10—Ø2.5 mm, 10.5 mm Long Ceramic Ferrule for MM Fiber, Ø340 µm bore size, ThorLabs, Newton, NJ, USA) were used. As a fiberoptic FT300UMT was implemented (0.39 NA, Ø300 µm Core Multimode Optical Fiber, High OH for 300—1200 nm, ThorLabs, Newton, NJ, USA). The end of the fiberoptics was polished and then glued to the ceramic ferrule. After virus/saline injection, the ceramic ferrule with fiber optics was implanted above the hippocampal neurons. After 3 weeks, optogenetic stimulation with the protocols mentioned in Section 2.2 and Section 2.3 was applied.

### 4.5. Fixed Slices Preparation and Evaluation of Density and Morphology of Dendritic Spines

The preparation of fixed slices of the hippocampus brain region was performed after transcardial perfusion with 1.5% PFA (pH = 7.4) solution in PBS. Mice were anesthetized via urethane diluted in a 0.9% NaCl solution. Hippocampal slices, 36 microns in thickness, were obtained by means of Campden 5100 mz Vibrotome (Campden Instruments vibrating microtome Ltd., Loughborough, UK). Slices were kept in 0.5% PFA. For microscopy slices were glued with Aqua Poly/Mount (Polysciences, #18606, Warrington, PA, USA) on microscope slides (Heinz Herenz, # 1042050, Hamburg, Germany) and coverslips (Menzel Glaser, #1, Braunschweig, Germany). Images of secondary dendrites of hippocampal neurons were made by a ThorLabs confocal microscope with the following parameters: Olympus UPlanSApo ×100 lens (UPlanSApo, 100×/1.40 Oil, OLYMPUS, Tokyo, Japan), resolution 2048 × 2048, slice thickness 0.2 microns/pixel and 50 field size. No more than 4 dendrites were taken into statistics from 1 slice. In total, 15 dendrites from one hemisphere were obtained for each mouse. After that, secondary dendritic spines density and morphology were analyzed at Neuronstudio [114] in the ways described before [64,115]. Dendritic spine density was measured as the number of spines divided by the analyzed length of the dendrite.

### 4.6. Fear Conditioning Behavioral Testing

On day 1 a mouse is placed in the cage A for fear conditioning for 5 min, allowing it to freely explore the space without the influence of conditional and unconditional stimuli (sound, current). The door of the insulation cabin is tightly closed every time. After the test, the mouse returns to the home cage. On day 2, the mouse is placed in the installation cage for 2 min, and then a conditional stimulus is applied for 20 s (sound—500 Hz at 80 dB), after which an unconditional stimulus is applied for 1.5–2 s (current—500 µA). The actions are repeated once. After that, the mouse is given 1 min of rest, after which it is moved to the home cage. On day 3, changes in early long-term memory were determined. The mouse was placed in the same test cage as used during the training of associated fear, but no more training was conducted, and the level of fear for a specific situation associated with a previous negative experience was recorded. In the next step, changes were made to the test cage to create a new environment unfamiliar to mice and the initial level of fear for the new environment was recorded. After that, the sound was turned on, and the level of fear associated with the conditional signal was registered. A week later, on day 10, the same approach was used as in the third day to determine long-term memory, the results of which are presented in the paper as the most exposed in mice of 5xFAD line.

### 4.7. Morris Water Maze Behavioral Test

A round white pool (diameter of 1.50 m, height of 0.66 m and depth of 0.29 m) was filled with nontransparent water (21–23 °C). A round platform (diameter of 0.10 m, height of 0.28 m) was put in the pool and placed 1.5 cm below the surface of the nontransparent water. The mice were given four consecutive training days with four trials per day in which they could find the platform hidden in the SE quadrant under muddy water. To find the hidden platform of search, the animals should navigate by four black-and-white signs located in four intercardiac directions at a height of 0.10 m above the water surface [93]. The mice began their 90 s swim in four different cardinal directions, which were randomly assigned. The starting point positions were the same for all mice. During each trial, the mice had 90 s to find the hidden platform. If the mice could not find the platform within 90 s, the observer brought them to the platform and allowed them to rest on the platform for at least 15 s. Between each trial, the mice were given a break of approximately 20 min to dry in the separate heated cages. On the fifth day of behavioral test, the mice had only one 90 s trial without a platform being found. The tests were recorded using a VS 1304-1 video system with Gigabit Ethernet software (1000Base-T). The Any-maze software was used to estimate the number of successful trials and the average platform search latency during training sessions.

### 4.8. Western Blot Analysis

FVB female mice (*n* = 8) at the age of 3.5 months were anesthetized by urethane (150 mg/mL) and xylazine (0.64 mg/mL). After a control check of the depth of anesthesia, mice were perfused with saline for 5 min to ensure the absence of the blood in the brain. After this, mice were decapitated and the hippocampus was removed from each hemisphere. Then, each hippocampus was homogenized in RIPA lysis buffer (50 mM Tris-HCl, 150 mM NaCl, pH 7.5, 0.1% sodium dodecyl sulfate, 0.5% sodium deoxycholate, 1% NP40, 1 mM PMSF, protease (Sigma, #S8820, St. Louis, MO, USA) and phosphatase (Sigma, #P0044, St. Louis, MO, USA) inhibitors) overnight at +4 °C. Next, total hippocampal lysate was mixed with loading buffer and kept 5 min at 90 °C. The total hippocampal lysates were electrophoretically separated by size on sodium dodecyl sulphate (SDS) polyacrylamide gel. Separated proteins were immobilized to PVDF membranes (#IPVH00010, Merck Millipore, Carrigtwohill, Ireland) preliminarily activated in methanol (#67-56-1, Merck, Carrigtwohill, Ireland). Non-specific proteins located on the membrane were blocked via incubation of the membrane in 5% bovine serum albumin (Sigma, #A9430, St. Louis, MO, USA) diluted in TBST (tris-buffered saline and Tween 20: 50 MM Tris, 150 MM NaCl, 0.1% Tween 20, pH = 7.6). Proteins of interests were detected with anti-actin (1:2000, #MAB1501, Merck Millipore, Carrigtwohill, Ireland), anti-GAPDH (1:8000, #A19056, ABclonal, Düsseldorf, Germany), anti-GFAP (1:1000, #544701, San Diego, CA, USA), ant-EAAT2 (#bs-1751R, Bioss, Woburn, MA, USA) and horseradish peroxidase-conjugated anti-rabbit (1:2000, DAKO, P0448) and anti-mouse (1:2000, DAKO, #P0447) secondary antibodies that were diluted in 2.5% BSA in TBST. For the increase in horseradish peroxidase luminescence, the buffer for enhanced chemiluminescence (ECL) was used (9 mL ddH_2_O, 1.5 mL 1 M Tris-HCl (pH = 8.8), as were 50 µL luminol (44 mg/mL), 22 µL paracumarate (15 mg/mL) and 70 µl 3% H_2_O_2_). Then, enhanced luminescence was recorded on X-ray film (#CP-BU M, AGFA, Mortsel, Belgium). Recorded luminescence was analyzed by ImageJ.

### 4.9. Statistical Analysis

All the data presented in the paper were verified in terms of the homogeneity of the sample using Bartlett’s test and they passed the test. Then, all the compared groups were checked in terms of normality by the Shapiro–Wilk test. Based on the obtained test results, data were compared using Student *t* tests or Mann–Whitney tests for paired comparison and via 2-way ANOVA test following Tukey tests or Kruskal–Wallis tests, with multiple-comparison Dunn’s tests used for multiple comparisons. Statistically significant differences were considered *p* < 0.05. All the data presented in the pictures and in text are shown as mean ± the standard error of the mean.

## Data Availability

The presented data that is shown in this study is available by a request from the corresponding author.
